# Effect of Proactive Personality on Career Decidedness: The Mediating Role of Career Exploration and the Moderating Role of Anticipated Regret

**DOI:** 10.3389/fpsyg.2021.763224

**Published:** 2021-11-16

**Authors:** Xuan Yu, Nan Luo, Xuhong Liu, Haihong Li, Ling Li, Yuanfei Mei

**Affiliations:** ^1^School of Life Science and Technology, University of Electronic Science and Technology, Chengdu, China; ^2^School of Business Administration, Chongqing Technology and Business University, Chongqing, China; ^3^Department of Police Management, Sichuan Police College, Luzhou, China; ^4^School of Management Science and Engineering, Chongqing Technology and Business University, Chongqing, China

**Keywords:** proactive personality, career exploration, career decidedness, anticipated regret, college students

## Abstract

In order to deepen the understanding of the personality antecedents of students’ career decidedness, this study, based on social cognitive career theory and career development theory, examined the effect of proactive personality on career decidedness as well as the mediating mechanism and moderating factors in this process. The results of the survey of 783 Chinese college students showed that proactive personality was positively related to career decidedness *via* career exploration, and anticipated regret moderated the relationship between proactive personality and career exploration significantly. Both theoretical and practical implications are discussed at the end of this research.

## Introduction

As college students are facing the transition from school to work, they need to make career-related preparations, which requires them to proactively carry out career management (Hirschi et al., 2013). Career decidedness refers to the level of certainty with respect to a particular career choice or career related decision (Gordon, 1981; Restubog et al., 2010). Previous study has shown that when students have a higher level of career decidedness, they are more motivated to seek internships and are more likely to succeed in securing internships (Hirschi and Läge, 2008). Given the critical role of career decidedness, we attempt to identify which groups of people show higher career decidedness.

So far, the Big Five model of personality has been proved to be the antecedents of career decidedness, but its effect size is not very large. Therefore, the researchers also pointed out that we should explore more personality antecedents of career decidedness (Lounsbury et al., 2005). Proactive personality is the dispositional basis for individuals to take proactive actions (Bateman and Crant, 1993), and researchers believe that it is an effective complement to the existing personality theories, because compared with the Big Five model of personality, proactive personality is more specifically related to personal career development (Major et al., 2006), Specifically, previous studies have shown that proactive personality can improve students’ career decision self-efficacy (Preston and Salim, 2019), and negatively predict their career decision-making difficulties (He et al., 2021). Therefore, we will examine the impact proactive personality on college students’ career decidedness by empirical method.

As is all we known, deciding on a career is a complex process for many students, because it is influenced by many personal and background factors (Praskova et al., 2015). Since the effect of personality on career decidedness is modest, we can also explore other variables, such as career exploration to explain variation in career decidedness (Lounsbury et al., 2005). Career exploration, as an individual’s proactive career behavior, can help individuals acquire knowledge about themselves and their environment, and improve their commitment to their goals (Jiang et al., 2019), which may help to enhance the level of students’ career decidedness. Moreover, personality traits are difficult to change and finding more mediating mechanisms can increase our chances of intervention in counseling (Shrout and Bolger, 2002), so we attempt to check the mediating role of career exploration to increase the possibility for interventions.

Besides, according to social cognitive career theory (SCCT, Lent et al., 2002), outcome expectation plays an important role in people’s career development and is the key mechanism for individuals to exert their initiative. Anticipatory regret is a negative emotional reaction that comes from comparing the expected result of not taking action with the expected result of taking action (Loewenstein and Lerner, 2003; Neneh, 2019). And as a self-directed outcome expectation, anticipated regret will affect the individual’s commitment to behaviors (Ajzen, 2011), which allows the proactive individuals to engage in more subsequent behaviors (Abraham and Sheeran, 2003). Therefore, this study will examine the role of anticipated regret between proactive personality and career exploration.

All above, the impact of proactive personality on career decidedness is the primary concern of this study. In addition, the mediating role of career exploration and the moderating role of anticipated regret in this process are also included in our research. Thus, according to proactive personality theory and SCCT, we conducted a survey among Chinese college students to test our ideas.

### Theoretical Background and Hypothesis

#### Proactive Personality and Career Decidedness

Proactive personality is a disposition factor that usually represents a relatively stable tendency to change the environment (Bateman and Crant, 1993). Individuals with this personality are not willing to be constrained by the force of the situation and show higher initiative (Crant, 2000). According to SCCT (Lent et al., 2002), one of the antecedents of self-efficacy belief is personality trait, specifically, the formation of self-efficacy belief partially depends on proactive tendency (Hirschi et al., 2013). At the same time, career decision self-efficacy (i.e., self-efficacy in career decision-making process) is considered to be a powerful predictor of career decidedness (Penn and Lent, 2018). Following this logic, we believe that students with proactive personality are more likely to possess career decision-making self-efficacy, thus enhancing their career decidedness.

Besides, we can learn the relationship between proactive personality on career decidedness from the existing empirical researches. The researchers have pointed out that proactive personality has a positive impact on conscientiousness and openness (Major et al., 2006; Fuller and Marler, 2009). Meanwhile, students get higher scores on conscientiousness and openness may have been more inclined to make a definite career choice (Shafer, 2000; Lounsbury et al., 2005). Based on the above discussion, there is a positive relationship between proactive personality and career decidedness. Therefore, we formulated our first hypothesis:

*Hypothesis 1:* proactive personality will be positively associated with career decidedness.

### The Mediating Role of Career Exploration

Career exploration is a proactive career management behavior (Hirschi et al., 2013), which is the action taken by individuals to gather information relevant to their careers, including self-exploration and environmental exploration (Zikic and Klehe, 2006). According to the integrative model developed by Crant (2000), proactive personality is seen as a vital antecedent to proactive behavior in more specific contexts, as it allows individuals to take initiative to change the environment (Crant, 2000; Fuller and Marler, 2009). So, students with proactive personality may explore themselves and/or the environment in an intended and systematic fashion (Stumpf et al., 1983). Existing studies have proved that proactive personality can influence career exploration through proximal construct such as self-efficacy (Hirschi et al., 2013). At the same time, through empirical tests and quasi-experimental methods, existing studies have proved that career exploration has a positive impact on career decidedness (Hirschi et al., 2011; Cheung and Jin, 2015). Besides, career exploration has been found to reduce career indecision (Park et al., 2016), which is view as inversely related to career decidedness (Leong and Chervinko, 1996).

Furthermore, according to career development theory, exploring and choosing a certain career are major tasks for college students (Emmanuelle, 2009), and individual initiative plays an important role in this process (Hirschi et al., 2013), while proactive personality reflects the tendency of individuals to take actions to identify external opportunities and change the environment (Bateman and Crant, 1993). As discussed before, proactive personality will enable students to carry out active career exploration so as to increase their ability to choose a specific career and their firmness to choose a career (Super and Kidd, 1979), which is career decidedness. Moreover, one research found career exploration serves as a mediator between proactive personality and future work self (Cai et al., 2015), while career decidedness can be seen as representing self-clarity regarding one’s future career development (Hirschi, 2014). To some extent, we think that the future work self and career decidedness have resemblance. Similarly, we speculate that career exploration may also play a mediating role in the relationship between proactive personality and career decidedness.

*Hypothesis 2:* Career exploration plays a mediating role between proactive personality and career decidedness.

### The Moderating Role of Anticipated Regret

Anticipatory regret is a negative emotional reaction that comes from comparing the expected result of not taking action with the expected result of taking action (Loewenstein and Lerner, 2003). That is, emotional reactions to possible outcomes also influence people’s decisions, and anticipated regret leads decision-makers to make more rational decisions (Zeelenberg, 1999). Given that career exploration is a rational decision for students as career exploration can help students gain useful information (Stumpf et al., 1983), so college students with proactive personality will conduct more career exploration under the influence of anticipated regret.

The regret regulation theory holds that the tendency to avoid negative emotions such as regret is an important determinant of human decision making (Zeelenberg and Pieters, 2007). When individuals with proactive personality that it is regrettable not to conduct career exploration, they will be involved in more career exploration as anticipated regret encourages people to take action to avoid negative emotions (Neneh, 2019). By contrast, although proactive personality to some extent represents people’s intention to take action (Antonacopoulou, 2000), individuals with proactive will not engage in more career exploration activities if they do not have an emotional reaction of anticipated expected regret from inaction, this is because these people are not strongly motivated to translate their intentions into behaviors (Abraham and Sheeran, 2003). Therefore, we believe that proactive personality has a stronger positive impact on career exploration with the increase of the level of anticipated regret, while the positive effect is weakened when the level of anticipated regret decreases.

*Hypothesis 3:* Anticipated regret will positively moderate the effect proactive personality has on career exploration.

**Figure 1:**
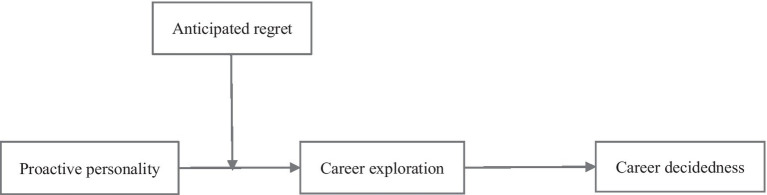
Proposed model of the study.

Figure 1 depicts our theoretical model.

## Materials and Methods

### Participants and Procedures

A cluster random sampling method was used to select 1,000 students from a college in Chongqing, China. In order to prevent the deviation of homologous methods, proactive personality, career exploration, anticipated regret and career decidedness were measured at different time points over a three-week period. At the first time point (T1), date about personal information was collected, and the proactive personality was measured in turn. A week later (T2), career exploration and anticipated regret were measured. Finally, career decidedness was measured in the third week (T3). Besides, this research has set up one item separately: the last four digits of the mobile phone number, so that the data corresponding to the above variables can be effectively matched.

SPSS 21.0 and AMOS 17.0 are used to analyze and process the data. The hierarchical regression method was used to analyze the main effect and the moderating effect, Hayes’ process macro plug-in was used to test the mediating effect (Hayes, 2015). At the same time, structural equation model was used to test the scale structure validity and path coefficient.

A total of 1,000 complete questionnaires were obtained by matching the last four digits of the mobile phone number, after incomplete questionnaires had been excluded, there were 783 (78.30%) valid responses. The characteristics of the sample data are shown in Table 1.

**Table 1:** Demographic information of participants.

		*N*	Proportion (%)
Gender	Male	184	23.5
	Female	599	76.5
Age	Under 18	73	9.3
	Between 19 and 20	449	57.4
	21 and above	261	33.3
Nationality	Han nationality	705	90.0
	Other ethnic minorities	78	10.0
Grade	Freshmen	333	42.5
	Sophomores	183	23.4
	Juniors	244	31.2
	Seniors	23	2.9
Major	Management	504	64.4
	Pedagogy	121	15.5
	Literature	84	10.7
	Economics	41	5.2
	Law	3	0.4
	Art	1	0.1
Parents’ education	Junior high school or below	482	61.6
	Senior high school, technical secondary school or technical school	188	24.0
	Junior college	54	6.9
	Undergraduate	55	7.0
	Postgraduate	4	0.5
Parent’s occupation	Private business owners/self-employed, unemployed, self-employed, agricultural workers and others employed	635	81.1
	Staff and workers of foreign enterprises and ordinary enterprises	85	10.9
	Staff and cadres of government organs or public institutions and others	63	8.0
Earlier participation in ECAs	Yes	160	20.4
	No	623	79.6
The number of ECAs (Sports/Drama/Dance/student newspaper/computer club, etc.)	Did not participate in any activities	44	27.7
	Participated in one activity	58	36.5
	Participated in two activities	44	27.3
	Participated in three activities	10	6.0
	Participated in four activities	4	2.4
Part-time hours per week	Less than 10h	669	85.4
	Between 10 and 20h	98	12.5
	More than 20h	16	2.0

*N=783*.

### Measures

In this study, we adopted the mature Western scales to measure the variables. For ensuring the consistency and applicability of the English scale in the Chinese context, the author conducted a translation-back translation procedure (Brislin, 1986). Before the formal investigation, a preliminary test was conducted on 15 college students, and the items were modified according to their feedback.

#### Proactive Personality

Proactive personality was measured with a 10-items scale developed by Li et al. (2014). Responses were on a five-point Likert scale ranging from 1 (strongly disagree) to 5 (strongly agree). A sample item was: “If I believe in an idea, there is nothing that will stop me from making it happen.” Cronbach’s alpha for this scale was 0.896.

#### Career Exploration

Career exploration was measured with an 11-items scale developed by Stumpf et al. (1983), which includes two dimensions: environmental exploration (e.g., “Investigated career possibilities”) and self-exploration (e.g., “Focused my thoughts on me as a person”). Participants were asked to “what extent have you behaved in the following ways over the last 3months?” from 1 (little) to 5 (a great deal). Cronbach’s alpha for environmental exploration subscale and self-exploration subscale were 0.82 and 0.76, respectively. Drawing on previous studies (e.g., Noe and Wilk, 1993; Cai et al., 2015), we did not distinguish the two dimensions of career exploration in our study, and took them as a whole indicator. Cronbach’s alpha was 0.884.

#### Career Decidedness

Career decidedness was measured with a six-item scale developed by Lounsbury et al. (2005). Responses were on a five-point Likert scale ranging from 1 (strongly disagree) to 5 (strongly agree). A sample item was: “I am sure about what I eventually want to do for a living.” Cronbach’s alpha for this scale was 0.878.

#### Anticipated Regret

Anticipated regret was measured with a two-item scale developed from Abraham and Sheeran (2003), and some words and sentences are modified according to our research background. Responses were on an eleven-point Likert scale ranging from 1 (definitely no) to 11 (definitely yes). A sample item was: “I would regret not pursuing my dream career within 12months of leaving college.” Cronbach’s alpha for this scale was 0.711.

#### Control Variables

The control variables were selected based on previous studies related to career exploration or career decidedness and the theory of planned behavior. These include gender, age, nationality, grade, major, parents’ education, parent’s occupation, earlier participation in ECAs, the number of ECAs, part-time hours per week, perceived behavioral control, locus of control, neuroticism.

Based on previous studies, firstly, we collected the general demographic information about the participants, including gender, age, nationality, grade and major (Neice and Bradley, 1979; Peter Eveland et al., 1998; Denault et al., 2019). Second, students’ experiences can also influence their career exploration behavior or career decidedness, such as earlier participation in ECAs, the number of ECAs and part-time hours per week (Denault et al., 2019). Third, some personality traits can also influence an individual’s level of career decidedness. Previous studies have shown that the internal locus of control makes people more determined about their career decisions (Cellini and Kantorowski, 1984). Besides, neuroticism is negatively related to career decidedness (Lounsbury et al., 2005). Thus, locus of control and neuroticism are considered to be related to career decision-making. Lastly, according to the theory of planned behavior, perceived behavioral control is considered as another factor affecting behavior in addition to intention (Ajzen, 1991). Moreover, existing studies have confirmed the role of perceived behavioral control in enhancing students’ decidedness in starting their entrepreneurial career, so, we speculate that it will affect students’ career decidedness. The above three constructs (locus of control, and neuroticism, perceptual behavioral control) were all measured using multiple items. Specifically, locus of control was measured with four items adapted from Shirokova et al. (2016). A sample item was: “When I make plans, I am almost certain to make them work.” Neuroticism was measured with three items developed by Hahn et al. (2012). A sample item was “gets nervous easily.” Perceived behavioral control was measured with four items adapted from Kautonen et al. (2015). A sample item was “It would be easy for me to make a career decision.” Lastly, according to the research of Denault et al. (2019), we also take the parents’ education level and occupation status as the control variables.

## Results

### Descriptive Statistics

Table 2 presents the descriptive statistics and correlations for the study variables. Proactive personality correlated moderately with career exploration (*r*=0.357, *p*<0.01) and career decidedness (*r*=0.424, *p*<0.01), and slightly with anticipated regret (*r*=0.194, *p*<0.01). Career exploration correlated slightly with anticipated regret (*r*=0.179, *p*<0.05) and correlated moderately with career decidedness (*r*=0.397, *p*<0.01). Besides, anticipated regret correlated slightly with career decidedness (*r*=0.127, *p*<0.05).

**Table 2:** Descriptive statistics and correlations of study variables.

S. no.	Variables	*M*	*SD*	1	2	3	4
1.	Proactive personality	3.466	0.547				
2.	Career exploration	3.069	0.611	0.357^**^			
3.	Anticipated regret	3.970	0.792	0.194^**^	0.179^**^		
4.	Career decidedness	3.209	0.625	0.424^**^	0.397^**^	0.127^**^	–
5.	Gender	1.770	0.424	−0.038	−0.085^*^	−0.023	−0.037
6.	Age	2.260	0.639	0.013	−0.037	0.032	−0.024
7.	Nationality	1.110	0.371	−0.025	−0.016	−0.002	−0.024
8.	Grade	1.950	0.923	0.011	0.002	0.052	−0.036
9.	Major	6.330	2.383	−0.012	−0.079^*^	−0.103^**^	−0.013
10.	Parents’ education	1.610	0.927	0.016	0.067	−0.006	0.013
11.	Parent’s occupation	1.270	0.599	0.034	0.045	0.024	−0.014
12.	Earlier participation in ECAs	0.800	0.403	0.119^**^	0.111^**^	0.115^**^	0.134^**^
13.	The number of ECAs	1.190	0.987	0.109^**^	0.100^**^	0.039	0.048
14.	Part-time hours per week	1.170	0.424	0.006	0.065	−0.010	0.003
15.	Perceived behavioral control	2.908	0.713	0.231^**^	0.269^**^	0.106^**^	0.276^**^
16.	Locus of control	3.205	0.551	0.286^**^	0.289^**^	0.229^**^	0.317^**^
17.	Neuroticism	4.597	0.879	0.024	−0.090^*^	0.163^**^	−0.096^**^

*N=783*,

* *p<0.05*,

** *p<0.01*.

The results of Harman single factor test showed that the variance explanation percentage of the first common factor was 29.527%, so it can be considered that there is no serious common method bias in this scale. Table 3 presents the structural validity of the scale. Since expected regret consists of two items, principal component analysis was adopted, KMO value is 0.602, Bartlett sphericity test significance is 0.000, and factor load values are all greater than 0.8. The results showed that the structure validity of each scale was good.

**Table 3:** Structure validity.

Variables	*AVE*	*CR*	*X* ^2^ */df*	NFI	CFI	RMSEA
Proactive personality	0.515	0.914	2.595	0.982	0.989	0.045
Career exploration	0.531	0.925	2.418	0.983	0.990	0.043
Career decidedness	0.556	0.882	3.092	0.989	0.992	0.052

### Test of Mediation

Table 4 presents the finding of the main effect test and mediation test. Proactive personality is positively correlated with career exploration (*B*=0.28, *SE*=0.03, *t*=8.18, *p*<0.001, *LLCI*=0.22, *ULCI*=0.35) and career decidedness (*B*=0.32, *SE*=0.04, *t*=8.47, *p*<0.001, *LLCI*=0.24, *ULCI*=0.39), which supports for hypothesis 1; Career exploration is positively correlated with career decidedness (*B*=0.26, *SE*=0.04, *t*=6.88, *p*<0.001, *LLCI*=0.18, *ULCI*=0.33) as well.

**Table 4:** Results of mediation analysis.

Antecedents	Career exploration						Career decidedness					
	B	SE	T	LLCI	ULCI	R^2^	B	SE	T	LLCI	ULCI	R^2^
						0.21^***^						0.29^***^
Constant	−0.61	0.36	−1.70	−1.32	0.10		−0.56	0.37	−1.49	−1.29	0.18	
Proactive personality	0.28	0.03	8.18^***^	0.22	0.35		0.32	0.04	8.47^***^	0.24	0.39	
Career exploration	–	–	–	–	–		0.26	0.04	6.88^***^	0.18	0.33	
**Control variables**												
Gender	−0.08	0.07	−1.18	−0.23	0.06		0.00	0.07	0.02	−0.14	0.15	
Age	−0.09	0.06	−1.43	−0.22	0.03		0.02	0.07	0.31	−0.11	0.15	
Nationality	−0.02	0.08	−0.21	−0.17	0.14		−0.03	0.08	−0.38	−0.19	0.13	
Grade	0.07	0.05	1.51	−0.02	0.16		−0.05	0.05	−1.04	−0.14	0.04	
Major	−0.03	0.01	−1.91	−0.05	0.00		0.01	0.01	0.57	−0.02	0.03	
Parents’ education	0.03	0.04	0.85	−0.04	0.11		0.01	0.04	0.22	−0.07	0.09	
Parent’s occupation	0.01	0.06	0.25	−0.10	0.13		−0.05	0.06	−0.90	−0.18	0.07	
Earlier participation in ECAs	0.09	0.08	1.23	−0.06	0.24		0.17	0.08	2.15^*^	0.01	0.32	
The number of ECAs	0.04	0.03	1.35	−0.02	0.10		−0.02	0.03	−0.62	−0.08	0.04	
Part-time hours per week	0.05	0.07	0.75	−0.09	0.19		−0.09	0.07	−1.20	−0.23	0.06	
Perceived behavioral control	0.16	0.05	3.11^***^	0.06	0.25		0.09	0.05	1.69	−0.01	0.19	
Locus of control	0.22	0.07	3.31^***^	0.09	0.34		0.23	0.07	3.47^***^	0.10	0.37	
Neuroticism	−0.08	0.03	−2.39^*^	−0.15	−0.01		−0.08	0.04	−2.29^*^	−0.15	−0.01	
Predictor								Effect	SE	LLCI	ULCI	
Direct effects (Proactive personality→Career decidedness)								0.32	0.04	0.24	0.39	
Indirect effects (Proactive personality→Career exploration→Career decidedness)								0.07	0.01	0.05	0.10	
Total effect (Proactive personality→Career decidedness)								0.39	0.04	0.32	0.46	

*N=783*,

* *p<0.05*,

*** *p<0.001*.

This study calculates bias-corrected bootstrapped 95% confidence intervals (using 5,000 bootstrap samples) for indirect effects of proactive personality on career decidedness through career exploration (as shown in Table 4). The direct effects of proactive personality on career decidedness is significant (*B*=0.32, *SE*=0.04, *LLCI*=0.24, *ULCI*=0.39), the indirect effects of proactive personality on career decidedness through career exploration is significant as well (*B*=0.07, *SE*=0.01, *LLCI*=0.05, *ULCI*=0.10), and the total effects of proactive personality on career decidedness (*B*=0.39, *SE*=0.04, *LLCI*=0.32, *ULCI*=0.46) is significant. That is, the positive relationship between proactive personality and career decidedness is mediated by career exploration. Hypothesis 2 is verified.

### Test of the Moderation

**Figure 2:**
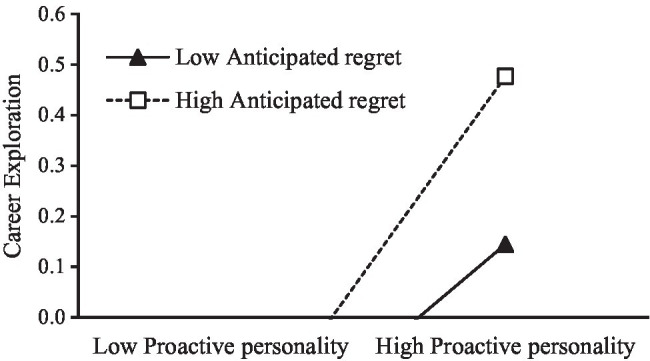
Interactive effect of proactive personality and anticipated regret on career exploration.

**Figure 3:**
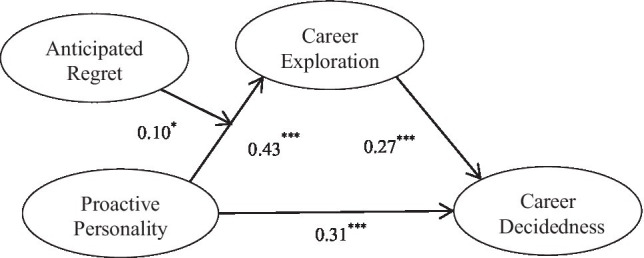
Theoretical research model with standard coefficients. *p<0.05, ***p<0.001.

As shown in Table 5, the interaction of proactive personality and anticipated regret significantly positively affects career exploration (*B*=0.09, *SE*=0.04, *t*=2.04, *p*<0.05, *LLCI*=0.01, *ULCI*=0.17), indicating that anticipated regret positively moderates the relationship between proactive personality and career exploration, and hypothesis 3 is verified. In addition, the simple slope analysis method is adopted to take one standard deviation from the mean value of anticipated regret and divide the sample data into two groups: the high anticipated regret group and the low anticipated regret group, and regression is conducted in the two groups, respectively. It can be seen that the effects of proactive personality on career exploration with different anticipated regret levels is different (as shown in Figure 2). On the high level of anticipated regret, proactive personality of college students has a greater impact on career exploration (*B*=0.35, *t*=6.88, *p*<0.001); on the low level of anticipated regret, the influence of college students’ proactive personality on career exploration is relatively smaller (*B*=0.22, *t*=4.83, *p*<0.001). Anticipated regret can stimulate proactive personality students to engage in career exploration behaviors and thus enhance career decidedness (see Figure 3). Therefore, hypothesis 3 is again verified.

**Table 5:** Results of moderation analysis.

Antecedents	Career exploration						Career decidedness					
	B	SE	T	LLCI	ULCI	R^2^	B	SE	T	LLCI	ULCI	R^2^
						0.21^***^						0.25^***^
Constant	−0.47	0.36	−1.29	−1.18	0.24		−0.66	0.39	−1.72	−1.43	0.10	
Proactive personality	0.26	0.04	7.27^***^	0.19	0.33		0.37	0.04	9.81^***^	0.30	0.44	
Anticipated regret	0.10	0.04	2.25^*^	0.01	0.18		0.02	0.05	0.35	−0.07	0.11	
PP X AR	0.09	0.04	2.04^*^	0.01	0.17		0.09	0.05	1.86	−0.01	0.18	
**Control variables**												
Gender	−0.08	0.07	−1.12	−0.22	0.06		−0.01	0.08	−0.18	−0.16	0.14	
Age	−0.09	0.06	−1.49	−0.22	0.03		−0.01	0.07	−0.09	−0.14	0.13	
Nationality	−0.02	0.08	−0.24	−0.18	0.14		−0.04	0.08	−0.43	−0.20	0.13	
Grade	0.07	0.04	1.45	−0.02	0.15		−0.03	0.05	−0.60	−0.12	0.07	
Major	−0.02	0.01	−1.72	−0.05	0.00		0.00	0.01	0.11	−0.03	0.03	
Parents’ education	0.04	0.04	1.15	−0.03	0.12		0.03	0.04	0.62	−0.06	0.11	
Parent’s occupation	0.00	0.06	0.03	−0.11	0.12		−0.06	0.06	−0.97	−0.18	0.06	
Earlier participation in ECAs	0.09	0.08	1.18	−0.06	0.24		0.20	0.08	2.46^**^	0.04	0.35	
The number of ECAs	0.04	0.03	1.17	−0.02	0.10		−0.01	0.03	−0.41	−0.08	0.05	
Part-time hours per week	0.06	0.07	0.78	−0.08	0.19		−0.08	0.08	−1.02	−0.23	0.07	
Perceived behavioral control	0.15	0.05	3.04^***^	0.05	0.25		0.13	0.05	2.35^*^	0.02	0.23	
Locus of control	0.18	0.07	2.72^**^	0.05	0.31		0.27	0.07	3.89^***^	0.14	0.41	
Neuroticism	−0.09	0.03	−2.72^**^	−0.16	−0.03		−0.10	0.04	−2.82^***^	−0.18	−0.03	

*PP, Proactive personality; AR, Anticipated regret*.

* *p<0.05*,

** *p<0.01*,

*** *p<0.001*.

## Discussion

### Theoretical Implications

Firstly, although previous study has shown that proactive personality can reduce students’ career indecision (Park, 2015), this study shows how proactive personality improves students’ career decidedness from a positive perspective. When exploring the relationship between personality and outcomes in a specific domain, basic personality traits (such as the Big Five) may be less valid than compound personalities associated with specific outcomes, such as proactive personality (Hough and Schneider, 1996; Hough, 2003). Proactive personality, in contrast to the Big Five personality traits, is particularly relevant to individuals’ career development and has been acknowledged to enrich existing personality theories (Major et al., 2006). In this study, proactive personality as a compound personality is proved to be a useful framework on the dispositional basis of career decidedness.

Second, this study demonstrates that the future career choice of individuals with proactive personality will be clearer and more committed through career exploration, which is consistent with the logic of Cai et al.’s (2015) study, that is, individuals with proactive personality will have a clearer cognition of future goals or orientations *via* career exploration. In addition, some previous studies believed that students engage in career exploration only when they possess a high level of career decidedness (Hirschi et al., 2011; Hirschi, 2014). However, this study shows that career exploration is the cause of career decidedness, in other words, instead of starting out with a clear career goal, students may discover themselves in the process of exploration, which ultimately clarify their future career.

Third, this study demonstrates the boundary condition (i.e., anticipated regret) that enhance the positive effect of proactive personality on career decidedness, which advance the career management literature insofar and makes us known more about the mechanism that influencing career exploration. Different from previous applications of anticipated regret, such as investment decisions (Bailey and Kinerson, 2005) and consumer behavior (Shih and Schau, 2011), we apply it into the career management process in order to broaden the horizon for future research. Besides, the existing studies on the individuals’ moderators between individual antecedents and career exploration mainly include traits or cognitive variables, such as self-construal (Hardin et al., 2006), proactive personality (Cai et al., 2015), and career calling (Praskova et al., 2015), while ignoring the influence of moods on behaviors (Jiang et al., 2019). In this study, we prove that anticipated regret will positively moderate the effect proactive personality has on career exploration, according to the result, we can understand when people with proactive personality are more likely to take actions.

### Practical Implications

These results are expected to provide a reference for counselors and students.

Firstly, based on our findings, we can identify students who may have difficulty making career decisions. As noted by Braden (1995), career counselors can design and implement differentiated interventions on the basis of understanding students’ personalities. By measuring individuals’ proactive personality, counselors may be able to predict which types of students are less likely to have high levels of career decidedness and tailor their career plans to their personality traits. For example, for individuals with low level of proactive personality, teachers or counselors can guide them to specify career planning and reward them when they complete certain tasks related to career, so as to stimulate their initiative to participate in career behaviors to enhance career decidedness.

Besides, since proactive personality is a stable dispositional basis, which should be hard to change (Seibert et al., 1999), the discovery of the mediating and moderating mechanisms gives us more opportunities for intervention. First, according to the results of our study, proactive personality positively associated with career decidedness *via* career exploration. For university career counselors, they need to encourage students to do more career exploration and schools should provide an environment that supports students to do more career exploration, such as setting up practical training courses to let students learn more career-related information and carrying out corresponding psychological tests, so that students can know more about themselves. Second, the results showed that anticipated regret plays a moderating role between proactive personality and career exploration. Furthermore, Individuals with proactivity have motivation to avoid their feelings of regret after making decisions, and their decisions are deeply influenced by regret aversion (Zeelenberg, 1999), so career counselor should help students realizing the possibility of anticipated regret, and subsequently persuade them to taken into account when making decisions so that promote students take actions. These measures are helpful for students to establish clear career goals and enhance career decidedness.

### Limitations and Future Research

The limitations of this study are as follows:

First of all, for the measurement of the outcome variable, we only collected the data of career exploration by means of self-report, but did not record students’ actually career exploration behavior. Therefore, this method may lead to common method bias and thus weaken the reliability of our research conclusions. So, we suggest that the relationship between proactive personality and career decidedness can be further examined by combining psychological experiments and case studies in future researches.

Besides, our research only explores the mechanism that affects students’ career decidedness from the individual’s perspective, while ignoring the influence of contextual factors. According to SCCT (Lent et al., 2002), the process of making career decisions is also inevitably influenced by contextual factors. Some studies have also confirmed parents’ behaviors, counselor’s functioning in the counseling context, etc. will affect students’ career exploration behaviors (Jiang et al., 2019), and then affect their career outcomes. Therefore, we could look for more antecedents of career decisions in the future researches.

Finally, although we measured variables at different time points, we did not measure all variables at each point in time. Hence, we cannot establish causality between the different measures absolutely. Especially, though the measuring career decidedness looks like the result of career exploration, they can also be considered a strengthening of career exploration in the past. Because some research shows the degree of goal clarity (Rogers et al., 2008) in relation to specific proactive career behaviors (e.g., career exploration), and career decidedness can be seen as representing clarity of self and goals regarding one’s career development (Hirschi, 2014). So, in other words, one’ career decidedness may also influence his engagement in career exploration. In the future, we can adopt longitudinal study design of growth curve analysis to collect data of all variables at different time points to study the dynamic process between career exploration and career decidedness (Raudenbush and Chan, 1992).

## Data Availability Statement

The raw data supporting the conclusions of this article will be made available by the authors, without undue reservation.

## Ethics Statement

Ethical review and approval was not required for the study on human participants in accordance with the local legislation and institutional requirements. The patients/participants provided their written informed consent to participate in this study.

## Author Contributions

NL wrote the manuscript and analyzed the data under the guidance of XY and LL. XL contributed to data analysis and editing of the manuscript. XY and HL contributed to study design and data collection. LL and YM contributed to study design and critical revisions. All authors contributed to the article and approved the submitted version.

## Funding

This work was supported by Youth Project of National Natural Science Foundation of China (71802033), Graduate Innovation Project of Chongqing (CYS21386), Key Research Base of Philosophy and Social Sciences in Sichuan Province-Social Governance Innovation Research Center Project (SHZLYB19009), Humanities and Social Sciences Research Project of Chongqing Education Commission (21SKGH125).

## Conflict of Interest

The authors declare that the research was conducted in the absence of any commercial or financial relationships that could be construed as a potential conflict of interest.

## Publisher’s Note

All claims expressed in this article are solely those of the authors and do not necessarily represent those of their affiliated organizations, or those of the publisher, the editors and the reviewers. Any product that may be evaluated in this article, or claim that may be made by its manufacturer, is not guaranteed or endorsed by the publisher.
